# Synthetic Collagen Hydrogels through Symmetric Self‐Assembly of Small Peptides

**DOI:** 10.1002/advs.202303228

**Published:** 2023-11-23

**Authors:** I. Caglar Tanrikulu, Lianna Dang, Lekha Nelavelli, Aubrey J. Ellison, Bradley D. Olsen, Song Jin, Ronald T. Raines

**Affiliations:** ^1^ Department of Chemistry Massachusetts Institute of Technology Cambridge MA 02139 USA; ^2^ Department of Chemistry University of Wisconsin–Madison Madison WI 53706 USA; ^3^ Department of Chemical Engineering Massachusetts Institute of Technology Cambridge MA 02139 USA

**Keywords:** biomaterials, collagen, peptide hydrogels, self‐assembly, symmetry

## Abstract

Animal‐sourced hydrogels, such as collagen, are widely used as extracellular‐matrix (ECM) mimics in tissue engineering but are plagued with problems of reproducibility, immunogenicity, and contamination. Synthetic, chemically defined hydrogels can avoid such issues. Despite the abundance of collagen in the ECM, synthetic collagen hydrogels are extremely rare due to design challenges brought on by the triple‐helical structure of collagen. Sticky‐ended symmetric self‐assembly (SESSA) overcomes these challenges by maximizing interactions between the strands of the triple helix, allowing the assembly of collagen‐mimetic peptides (CMPs) into robust synthetic collagen nanofibers. This optimization, however, also minimizes interfiber contacts. In this work, symmetric association states for the SESSA of short CMPs to probe their increased propensity for interfiber association are modelled. It is found that 33‐residue CMPs not only self‐assemble through sticky ends, but also form hydrogels. These self‐assemblies behave with remarkable consistency across multiple scales and present a clear link between their triple‐helical architecture and the properties of their hydrogels. The results show that SESSA is an effective and robust design methodology that enables the rational design of synthetic collagen hydrogels.

## Graphical abstract

Nanofibers produced by the sticky‐ended symmetric self‐assembly (SESSA) of 33‐residue collagen‐mimetic peptides (CMPs) exhibit higher‐order structures, consistent with molecular models, leading to “needles” and fibrils, and ultimately, a noncovalent hydrogel featuring the shortest CMP building blocks to date. Despite such complexity, both self‐assembly and hydrogelation can be linked directly to collagen triple‐helical structure, a first for such a material.

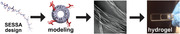

## 1 Introduction

Except for teeth, nails, and bone, the human body is a hydrogel.^[^ ^1^ ^]^ Hydrogels are hydrophilic polymer networks that can retain water at more than 100 times their own weight.^[^ ^2^ ^]^ As materials, hydrogels are commonly employed as extracellular matrix (ECM) mimics in tissue engineering and medicine, as their viscoelastic and mass–transfer properties and their high porosity resemble those of human tissues. Hydrogels that rely on protein or peptide networks are of special interest because they provide an expeditious interface with biology and allow cells to remodel their microenvironments.^[^ ^3^ ^]^

Collagen is the most abundant protein in animals and a dominant component of all connective tissues in humans.^[^ ^4^ ^]^ Because animal‐sourced collagen readily forms hydrogels (e.g., gelatin) and is easily available and inherently compatible with cell biology, collagen‐based hydrogels have become a common substrate for culturing mammalian cells.^[^ ^5^ ^]^ Their clinical use is, however, complicated by issues of reproducibility, immunogenicity, and contamination.^[^ ^5^ ^]^

The self‐assembly of peptides and proteins could overcome such limitations by allowing access to chemically defined protein hydrogels. Interactions between protein secondary structural units, such as α‐helices and β‐sheets, have enabled a selection of self‐assembling hydrogel designs.^[^ ^3^, ^6^ ^]^ Still, despite being an essential component of the ECM, the triple‐helical architecture of collagen has only been rarely used for the formation of hydrogels.

**Figure 1:**
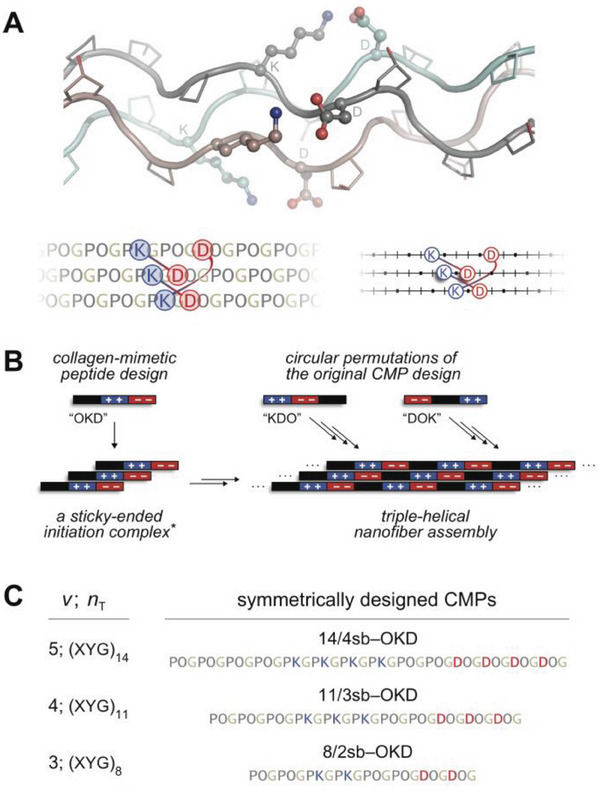
Design of sticky‐ended synthetic collagen self‐assemblies. A) Molecular structure of the collagen triple helix and depictions of axial salt bridges therein. A [(POG)_n_]_3_ triple helix model with a Lys···Asp salt bridge between each strand pair was built with PyMOL v1.8 based on coordinates from PDB entry 3u29, the X‐ray crystal structure of [(POG)_3_‐PKG‐DOG‐(POG)_3_]_3_ (top).^[^ ^14c^ ^]^ Identities of residues on the gray strand are marked next to their corresponding C^α^ atoms. Two alternative representations of this model highlight salt‐bridging residues with interconnected blue and red circles, whereas Pro/Hyp and Gly residues on a CMP are depicted simply as bars and dots, respectively, on a line (bottom). Top, middle, and bottom strands on the representations correspond to cyan, brown, and gray strands in the molecular model, respectively. B) A tri‐block CMP and its circular permutants produce the same pattern when assembled symmetrically. *Although a trimer is shown, features of the initiation complex are not clear. C) CMP designs for sticky‐ended symmetric self‐assembly discussed (14/4sb) or tested (11/3sb and 8/2sb) in this study, where, *n*_T_, the number of XYG blocks, is 3*v* − 1.

Collagens are characterized by their Xaa‐Yaa‐Gly tripeptide repeats, where Xaa and Yaa are commonly (2*S*)‐proline (Pro or P) and (2*S*,4*R*)−4‐hydroxyproline (Hyp or O), respectively, followed by an essential glycine residue (Gly or G) (**Figure** **1**A).^[^ ^7^ ^]^ This sequence favors a polyproline II‐type fold and enables the parallel association of three collagen strands that are staggered by a single residue into a triple helix. This association is largely driven by main‐chain–main‐chain contacts.^[^ ^4^, ^8^ ^]^ Whereas nature employs numerous chaperones, posttranslational modifications, and transport pathways to ensure correct strand association,^[^ ^9^ ^]^ the scarcity of sequence‐specific interstrand contacts complicates synthetic collagen self‐assembly design.

Synthetic collagen hydrogels commonly feature short, self‐assembling peptides that support the triple‐helical architecture (i.e., collagen‐mimetic peptides; CMPs), mostly as trimer‐forming scaffolds displaying functional groups that enable hydrogelation.^[^ ^10^ ^]^ These designs fail to capture the triple‐helical nanofibers that are characteristic of natural collagen. Hydrogelation of synthetic collagen nanofiber networks has been realized only a few times through the use of “sticky‐ended” self‐assembly. This mode of assembly pushes strands to associate with large offsets, exposing sticky ends that recruit more CMP building blocks to the growing triple helix (Figure 1B).^[^ ^11^ ^]^ In early designs, strand offsets were enforced by interstrand disulfide bridges, and gelation of the resulting self‐assemblies correlated well with triple‐helical structure.^[^ ^12^ ^]^ Self‐assembly of free strands was later achieved through the use of interstrand (2*S*)‐lysine (Lys or K)–(2*S*)‐aspartic acid (Asp or D) salt bridges.^[^ ^13^ ^]^ When formed, “axial” salt bridges between a Lys residue (at Yaa) and an Asp residue (at Xaa that is three residues away on a neighboring strand) boost triple‐helical stability and direct strand association (Figure 1A).^[^ ^14^ ^]^ By installing Lys···Asp salt bridges on an earlier 36‐residue CMP design,^[^ ^15^ ^]^ Hartgerink and coworkers created the tri‐block peptide F0, which is (PKG)_4_(POG)_4_(DOG)_4_, and documented its hierarchical assembly into triple‐helical nanofibers and hydrogels that are stable in a cell‐culture medium.^[^ ^13^ ^]^

Hierarchical self‐assembly of F0 into a hydrogel has been attributed to the availability of numerous unpaired Lys and Asp residues on self‐assembled triple‐helical nanofibers.^[^ ^13^ ^]^ These positively and negatively charged residues can facilitate interactions between nanofibers and drive the formation of higher‐order structures and hydrogels. It is difficult to delineate these interactions, however, because the F0 sequence allows for five distinct but energetically comparable triple‐helical association modes that produce different patterns of unpaired charges on the nanofiber surface.^[^ ^16^ ^]^ Interestingly, other CMPs with similar sequence motifs and assembly modes to F0 fail to form hydrogels. For example, shuffling the tri‐block organization of F0 allows self‐assembly, but not hydrogel formation.^[^ ^17^ ^]^ Further, circular permutants of F0, which are expected to assemble similarly (Figure 1B), form aggregates instead.^[^ ^17^ ^]^ This complexity obfuscates the mechanisms that enable the self‐assembly of F0 variants and the hydrogelation of F0, alike.

Sticky‐ended symmetric self‐assembly (SESSA) overcomes this challenge by maximizing the contribution of axial salt bridges to assembly thermostability through the use of symmetry. A perfect pairing of Lys and Asp residues allows CMP self‐assembly to proceed through a unique, maximally stabilized association state, producing a defined assembly. We recently applied SESSA to 42‐mer CMPs to obtain the 14/4sb series, peptides with 14 tripeptide repeats that donate and receive four axial salt bridges from neighboring CMPs upon assembly.^[^ ^16^ ^]^ Unlike F0, all 14/4sb circular permutants reliably self‐assemble into predictable and thermostable structures, elevating SESSA as a platform for the exploration of the self‐assembly and gelation behavior of synthetic collagens. The fine nanofiber networks formed by symmetrically designed 14/4sb peptides, however, failed to yield hydrogels.

SESSA minimizes the availability of free Lys and Asp residues on nanofiber assemblies in order to achieve perfect pairing. This parsimony could, however, impede higher‐order association and hydrogelation. We hypothesized that a symmetrically designed system that allows for the bundling of nanofibers might enable us to access hydrogels through SESSA. Here, we apply SESSA to short (33‐residue) CMPs, which we predict to better support triple‐helical bundling and hydrogel formation. By surveying symmetric CMP designs for both self‐assembly and hydrogelation, we identify the shortest known CMPs that self‐assemble through sticky ends, verify hydrogel formation, characterize rigorously those hydrogels, and link their material strength to the triple‐helical structure. Identical patterns of thermostability and nanostructure appear across symmetrically designed CMPs, which indicate a common assembly route and establish SESSA as a robust methodology for the rational design of synthetic collagen biomaterials.

## 2 Results

### 2.1 Peptide Design and Synthesis

SESSA improves CMP self‐assembly by providing a unique assembly route to triple–helix formation, which proceeds through the symmetric‐association state (SAS).^[^ ^16^ ^]^ This route ensures the symmetric arrangement of triblock CMPs and the perfect pairing of their Lys and Asp residues in the final assembly. For a CMP featuring *n*_T_ number of Xaa‐Yaa‐Gly tripeptide repeats, SAS requires a strand offset of *n*_T_ residues, and in the collagen context, this offset is possible only when *n*_T_ is not a multiple of three (*n*_T_ = 3*v* ± 1, where *v* ∈ **ℤ** ^+^). The 14/4sb set, explored in our previous work, represented the application of SESSA to a (POG)_14_ template (42 residues; *v*  =  5, *n*_T_ = 3*v* − 1 = 14). Of the resulting design, all three circular permutants tested—OKD, KDO, and DOK—yielded surprisingly thin (2–8 nm) and thermostable (*T*_m_ = 38–49 °C) nanofibers.^[^ ^16^ ^]^ Interestingly, however, these self‐assemblies did not produce hydrogels.

Commonly, the formation of nanofiber hydrogel networks relies on the extent of nanofiber entanglement and their cross‐linking. Although the molecular assembly models predicted by SESSA provide limited insight into entanglement behavior, they can provide valuable information on the availability of molecular contacts interconnecting nanofibers. For this, we explored peptides shorter than 42‐mers (*v* = 5), as these are more accessible than longer peptides in the 3*v* − 1 series (e.g., *v* = 6; 51‐mers). The next smaller SESSA‐compatible CMPs in this series after a 42‐mer are a 24‐mer (*v* = 3; *n*_T_ = 8) and a 33‐mer (*v* = 4; *n*_T_ = 11), and we focused our efforts on 33‐mers, due to the radically short size of the former (Figure 1C). Although significant reductions in peptide length are expected to diminish assembly thermostability, we were primarily interested in exploring any improvements a 33‐mer assembly might provide on the bundling of nanofibers.

**Figure 2:**
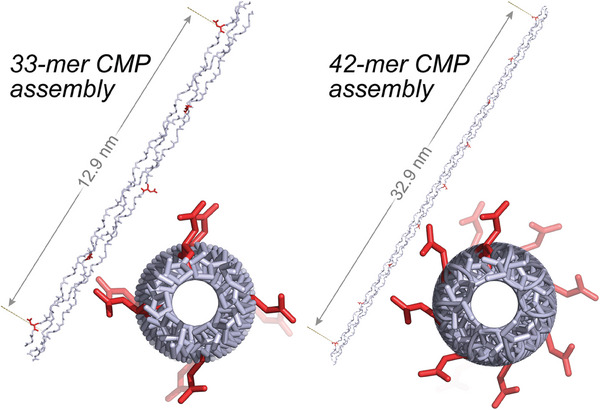
Comparison of molecular models for the symmetric self‐assembly of 33‐mer and 42‐mer CMPs into nanofibers. A single Asp residue is marked as red on each peptide in the assembly, and the orientation of this side chain is tracked along the assembly relative to the triple‐helical axis. The shortest distance between two such residues appearing on the same side of the triple‐helical assembly is reported. A top‐down view highlights differences in the distribution of Asp side chains around the triple helix. In contrast to one featuring a 42‐mer, the SESSA model of a 33‐residue CMP produces a regular arrangement of potential interaction partners along the triple‐helical axis and presents a geometry favorable for nanofiber association and bundling.

The stable association of extended triple helices into bundles requires repeating molecular contacts along the nanofiber–nanofiber interface. We, therefore, compared the availability of such periodic interactions along the triple‐helical axis in molecular SESSA models of 42‐mer and 33‐mer CMP self‐assemblies. For self‐assembled 33‐mers, a specific contact repeats once every 11 residues along the nanofiber with a ≈90° rotation around the helical axis (**Figure** **2**). Thus, an unsatisfied molecular contact, such as a charged side chain or terminus, reappears on the same face (i.e., ≈360° rotation) every ≈13 nm and could complement similarly interspaced contacts on a neighboring nanofiber. In contrast, an unpaired contact on 42‐mer CMP assemblies repeats every 14 residues with a ≈47° shift. These attributes lead to a long periodicity (33 nm) and a poor eclipse (≈16° offset), presenting a geometry that is suboptimal for bundle formation and contrasts the favorable geometry observed on 33‐mer SESSA models.

**Figure 3:**
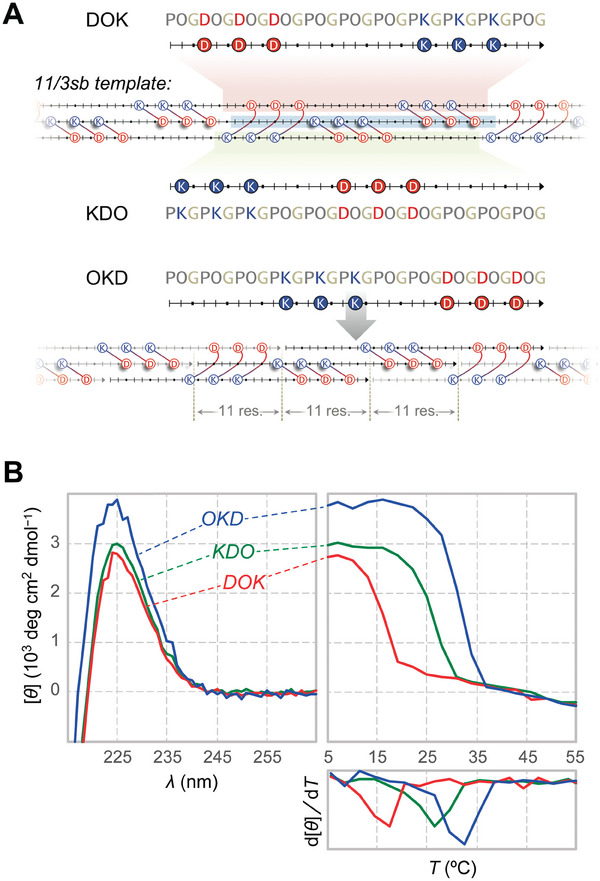
Self‐assembly of 11/3sb permutants. A) Symmetric assembly of DOK, KDO, and OKD permutants is presented on the 11/3sb template, highlighted in red, green, and blue, respectively. The uniform 11‐residue interstrand stagger required for symmetric assembly and the resulting perfect charge pairing common to all 11/3sb permutants are demonstrated with OKD. B) CD spectra and thermal denaturation curves confirm triple‐helical assembly for all 11/3sb permutants at 0.6 mg/mL. Samples were prepared in 10 mm sodium phosphate buffer, pH 7.0, unless noted otherwise.

To evaluate the self‐assembly and hydrogelation of symmetrically designed 33‐mer CMPs, we designed 33‐residue sequences compatible with the SESSA rules.^[^ ^16^ ^]^ Anticipating a significant loss of thermostability with a shorter peptide, we lowered the number of salt‐bridging pairs from four in 14/4sb to three to conserve stabilizing Pro‐Hyp‐Gly segments in the sequence,^[^ ^18^ ^]^ arriving at the 11/3sb design (Figure 1C). Additionally, we produced the 8/2sb design to test the lower CMP‐size limit among the 3*v* − 1 series of self‐assemblies. We synthesized three circular permutants of 11/3sb (OKD, KDO, and DOK; **Figure** **3**A) that represent three extremes for charge placement on a 33‐mer sequence, as well as 8/2sb‐OKD, through protocols established previously for CMP synthesis^[^ ^19^ ^]^ and purification (Figure S1 and S2A, Supporting Information).^[^ ^16^ ^]^ The resulting peptides are expected to associate with uniform 11‐ or 8‐residue sticky ends into symmetric self‐assemblies (Figures 3A and S2B, Supporting Information), and the OKD, KDO, and DOK permutations investigated are analogous to those tested previously for 14/4sb. For analysis, samples were prepared in 10 mm sodium phosphate buffer, pH 7.0,^[^ ^14b^ ^]^ and annealed over 4.25 h from 55 to 4 °C, where they remained for ≥48 h prior to data acquisition, unless stated otherwise.^[^ ^16^ ^]^

### 2.2 Solution‐Phase Characterization of CMP Self‐Assemblies

All 11/3sb variants display a strong circular dichroism (CD) signature for triple–helix formation at 226 nm that dissociates cooperatively in thermal denaturation experiments (Figure 3B), whereas 8/2sb‐OKD data indicate no triple–helix formation except in buffer–methanol mixtures (Figure S2C, Supporting Information). The thermostabilities of symmetric 33‐mer assemblies (*T*_m_ = 32, 27, and 17 °C for 0.6 mg/mL OKD, KDO, and DOK, respectively; **Table** **1**) are on par with or better than those from similar 36‐mer designs (*T*_m_ = 15–25 °C),^[^ ^17^ ^]^ making them the shortest peptides to support this mode of self‐assembly. Although the *T*_m_ values for 11/3sb permutants are markedly lower than those of their 14/4sb counterparts (*T*_m_ = 38–49 °C),^[^ ^16^ ^]^ assembly thermostability ranks as *T*_m_(OKD) > *T*_m_(KDO) > *T*_m_(DOK) across both series. Such congruency suggests similar factors impacting thermostability in both systems, despite differences in charge count and distribution, and CMP size.

**Table 1:** Values of *T*_m_ and nanofiber dimensions for 11/3sb‐derived CMPs.

11/3sb variant	*T*_m_ (°C)		Nanofiber width (nm) by TEM
	*I* = 20 mm	*I* = 200 mm	
OKD	32	26	11 ± 3^a)^
KDO	27	20	4 ± 1
DOK	17	12	3 ± 1
DOKctrl	n.d.^b)^	21	n.o.^c)^

^a)^ Dimension of OKD needles. Association of needles reveals fibrils that are ≈50–200 nm wide;

^b)^ *T*_m_ could not be determined for DOKctrl due to a shallow transition;

^c)^ Nanofibers were not observed for DOKctrl.

A strong collagen CD signal is necessary but not sufficient to establish SESSA. CMPs commonly form triple helices featuring three strands that associate with a single‐residue offset, displaying “blunt ends,” which may also lead to fibrillar structures.^[^ ^15^ ^]^ The thermostability of blunt‐ended triple helices is generally defined by their sequence composition.^[^ ^20^ ^]^ In contrast, the *n*_T_‐residue strand offsets required to form symmetric sticky ends necessitate strong, sequence‐specific interstrand interactions that stabilize the SAS. Symmetric association is only possible through the SAS, and any disruption to its stability can significantly impair SESSA.^[^ ^16^ ^]^ Thus, a sequence rearrangement on a symmetrically designed CMP would not significantly alter the stability of its blunt‐ended triple helices but could be detrimental for sticky‐ended association.

**Figure 4:**
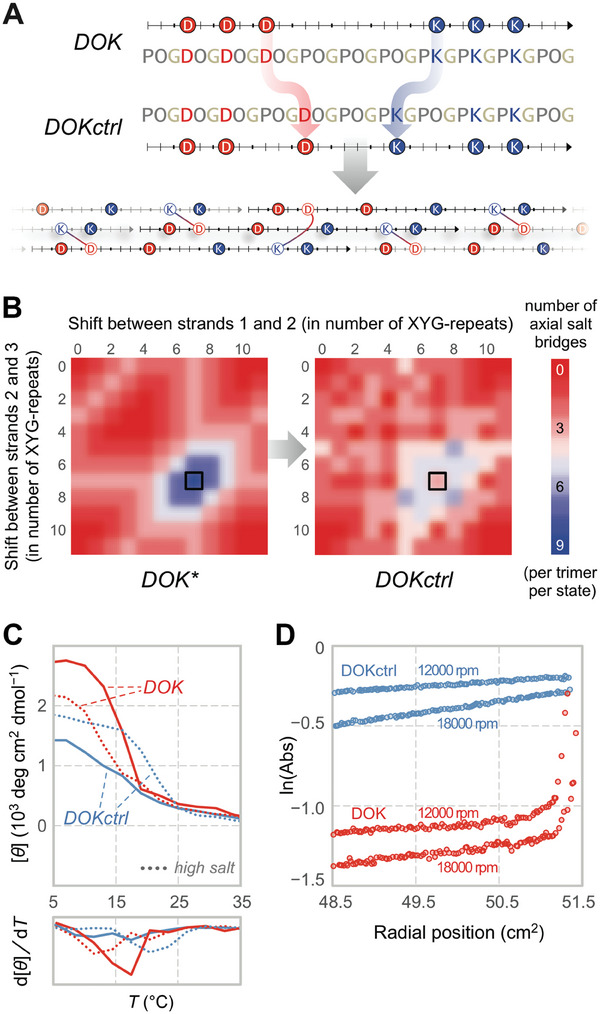
Disruption of the salt‐bridge network eliminates symmetric self‐assembly. A) A slight modification to the DOK sequence results in a peptide (DOKctrl) that is unable to establish ⅔ of the salt bridges accessible to its parent upon symmetric assembly. This change is reflected in the strand‐association landscape (SAL) for DOKctrl B). For a given CMP, SAL presents the number of axial salt bridges supported by every possible strand association leading to sticky‐ended self‐assembly into an extended triple helix. The symmetric association state (SAS; black borders) appears at tripeptide shifts of (7,7), which is equivalent to (−4,−4), and provides 9 and 3 axial salt bridges per trimer for DOK and DOKctrl, respectively. DOKctrl loses the well‐defined singular maximum of DOK at the SAS. *This landscape describes strand association for all 11/3sb permutants, as well as DOK. C) CD thermal denaturation curves for DOK and DOKctrl (0.6 mg/mL) at low (20 mm, solid line) and high ionic strength (200 mm, dotted line). D) Sedimentation equilibrium gradients for DOK and DOKctrl (0.3 mg/mL). The divergent impact of ionic strength on triple‐helicity and differences in assembly size both indicate dissimilar molecular organization when strand association is disrupted.

In order to dissect the assembly route for DOK, we constructed DOKctrl, a DOK variant with two residue swaps (**Figure** **4**A–B). This modification does not alter sequence composition but weakens the SAS by eliminating 2/3 of the axial salt bridges stabilizing it. Such a change would significantly impair self‐assembly only if DOK assembly proceeds through sticky‐ended association. Our data confirm this proposition. In contrast to DOK, DOKctrl exhibits a weak thermal transition at low ionic strength (*I* = 20 mm). The weakened Coulombic interactions induced by high ionic strength (*I* = 200 mm), however, cause both the *T*_m_ value and triple helicity of DOKctrl to increase dramatically (Figure 4C). This effect of ionic strength on DOKctrl association contrasts sharply with that on DOK and other assemblies (Figure S3) and is consistent with the formation of blunt‐ended DOKctrl homotrimers burdened with Coulombic repulsion. Sedimentation equilibrium experiments confirm the homotrimerization of DOKctrl (a monomer–trimer mixture) while revealing large DOK multimers (Figures 4D and S4, Supporting Information). Hence, the formation of the specific charge pairs required for SESSA is critical for DOK self‐assembly, mirroring our earlier results.^[^ ^16^ ^]^

### 2.3 Nanostructure Characterization

**Figure 5:**
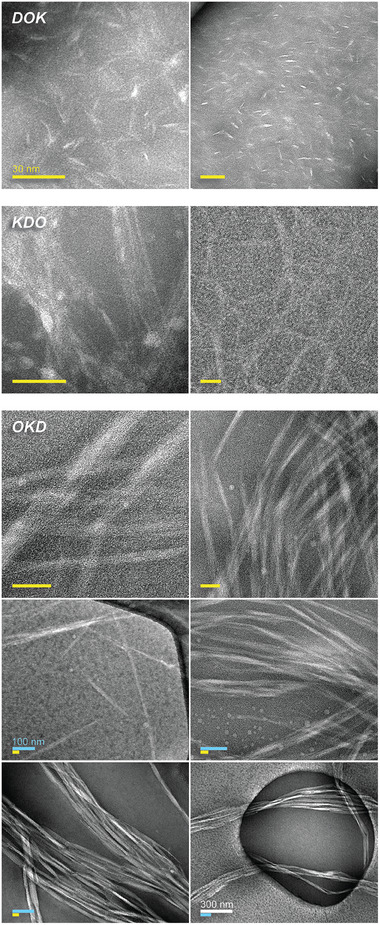
TEM images of self‐assembled 11/3sb permutants reveal nanofibers and nanofibers bundles. Whereas 11/3sb‐DOK and KDO assemble into nanofibers 3 ± 1 nm and 4 ± 1 nm wide, OKD assembles into 11 ± 3 nm thick needles. Hierarchical assembly of OKD nanofibers into fibrils is presented at various scales. OKD fibrils can reach a width of up to 0.2 µm. Scale bars indicate 30 nm (yellow), 100 nm (blue), or 300 nm (white).

Visualization of self‐assemblies by negative‐stained transmission electron microscopy (TEM) reveals the formation of thin nanofibers for all 11/3sb permutants (**Figure** **5**), consistent with our design objectives. The DOK and KDO nanofibers are thin (mean width ± SD of 3 ± 1 and 4 ± 1 nm, respectively; Table 1), but with slightly different morphologies and aspect ratios. DOK assemblies are relatively short (≈25 nm), whereas KDO nanofibers often appear in 20‐ to 100‐nm thick bundles that can reach 0.5 µm in length (Figure 5). In contrast, OKD assembles into needle‐like structures that are 11 ± 3 nm wide and 0.2–0.6 µm long, which are likely to be aligned bundles of extended triple‐helical self‐assemblies. “Needles” associate predominantly end‐to‐end, but also side‐to‐side, to form 50‐ to 200‐nm wide fibrils that commonly extend for multiple micrometers (Figure 5, OKD, bottom panels). Overall, despite their differing morphologies, all permutants based on the 11/3sb template form thin, soluble nanofibers, as expected of CMPs adhering to SESSA.

### 2.4 Rheology

**Figure 6:**
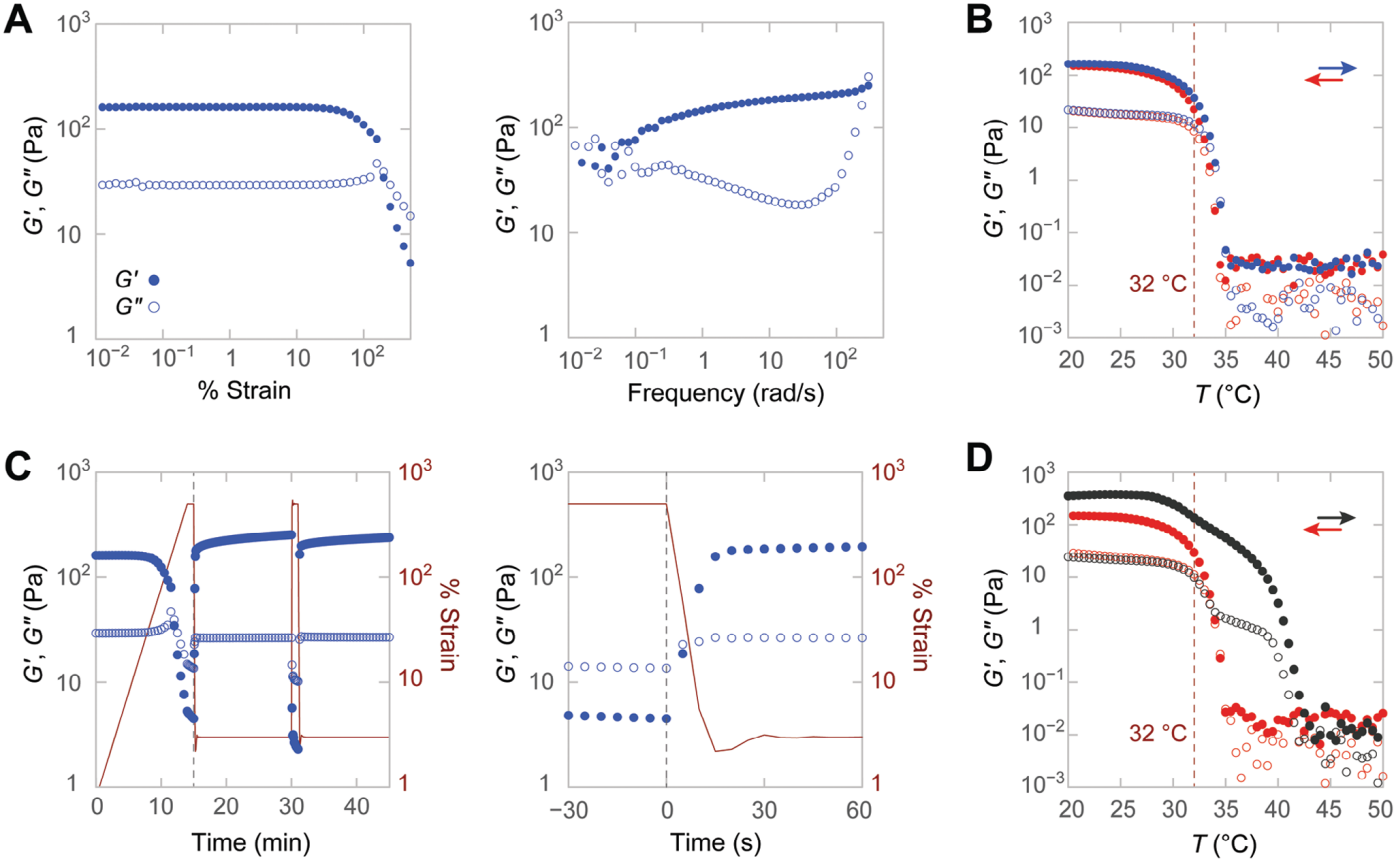
Rheology of 11/3sb‐OKD hydrogels. Closed (⬤) and open circles (○) report storage and loss moduli in all panels. A) Strain (at 3 rad/s) and frequency sweeps (at 1% strain) of a 1.0% w/v hydrogel. B) Temperature denaturation (blue) and subsequent annealing (red) of a 1.0% w/v OKD hydrogel reveal a transition profile consistent with CD data. The temperature response was monitored at 1% strain and 3 rad/s in all experiments. The value of *T*_m_ determined by CD spectroscopy is indicated (dashed line). C) Fast recovery of the hydrogel after 500% strain. Moduli (blue) and strain (red) are shown over time. The dashed line identifies an identical time point between the two panels. D) Temperature denaturation (black) and subsequent annealing (red) of a previously deformed 1.0% w/v OKD hydrogel highlight its elevated thermostability after deformation.

In further contrast to 14/4sb, the 11/3sb system allows for hydrogelation. The OKD permutant forms hydrogels at concentrations of 2 mg/mL (i.e., 0.2% w/v) or higher in 10 mm sodium phosphate buffer, pH 7.0. Heat denaturation and subsequent cooling indicate gelation within minutes. OKD gels at 0.5% and 1.0% w/v present storage moduli (*G*′ = ≈80 and ≈160 Pa, respectively) significantly higher than loss moduli (*G*′′ = ≈15 and ≈30 Pa) over a wide range of oscillation frequencies and amplitudes (**Figures** **6**A and S5, Supporting Information), which is consistent with hydrogel formation. These values match (at 0.5% w/v) or exceed (at 1.0% w/v) those obtained for Matrigel, a biologically sourced ECM mimic and common cell culture substrate (*G*′ = 55 Pa).^[^ ^21^ ^]^ Furthermore, OKD hydrogels are stable at temperatures up to 32 °C, after which the material transitions from a solid‐like to a liquid‐like state (Figure 6B). This transition is almost indistinguishable from the denaturation transition observed with CD spectroscopy (*T*_m_ = 32 °C; Figure 3A). Conversely, annealing OKD by cooling through its *T*_m_ value immediately restores the hydrogel with minimal hysteresis (Figure 6B). OKD hydrogels also form in cell culture medium (EBM‐2; *I* = ≈0.2 M) below 26 °C, which is the *T*_m_ value measured for OKD at *I* = 0.2 M via CD spectroscopy (Figure S6, Supporting Information). In contrast to previous work, where the loss of triple helicity accompanies a rising *G*′ value,^[^ ^13^ ^]^ our data point to a direct link between the triple‐helical structure and the material properties of OKD self‐assemblies.

**Figure 7:**
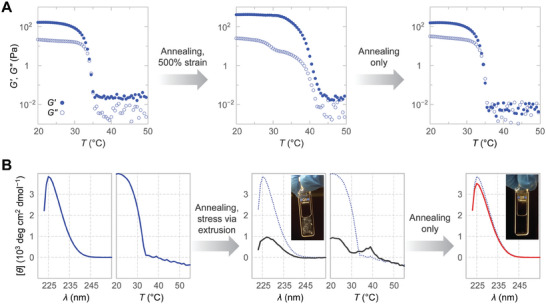
Impact of deformation on hydrogel rheology and triple‐helical self‐assembly. A) Altered temperature denaturation profile of a previously deformed 1.0% w/v hydrogel returns to its original form following annealing. The temperature response was monitored at 1% strain and 3 rad/s. Closed (⬤) and open circles (○) report storage and loss moduli. B) Extrusion of a 0.5% w/v hydrogel produces a white precipitate and reveals a second melting transition in CD thermal denaturation experiments, which is in agreement with rheology data. CD spectra and visual inspection reveal the restoration of the original sample upon annealing. The signal was monitored at 226 nm in all denaturation experiments. The initial denaturation curve and spectra are shown in all panels as a reference (dotted blue lines).

11/3sb‐OKD hydrogels recover remarkably fast after they are pushed to 500% oscillatory strain, which is beyond the end of the linear regime (≈30%) (Figure 6C). Both 0.5% and 1.0% w/v gels regain 90% of *G*′ within 20 s after strain application, even after repeated cycles of high‐strain deformation. Surprisingly, the application of stress doubles *G*′ and enhances thermostability so that prestressed gels maintain their structure up to 40 °C, which is well above their *T*_m_ value of 32 °C (Figure 6D). Cooling a prestressed gel from 55 °C restores the mechanical properties of the undeformed gel (**Figure** **7**A), whereas cooling from 45 °C partially maintains the elevated thermostability (Figure S7, Supporting Information).

To determine the role of triple‐helical structure in these transitions, we monitored triple helicity in stressed OKD hydrogels (Figure 7B). Stressing the gel via extrusion through a 22G needle resulted in a white precipitate, reducing the CD signal. Interestingly, subsequent thermal denaturation occurred with a transition corresponding to that observed on the rheometer near 40 °C, indicating that the triple‐helical structure is maintained up to this temperature. These triple helices are likely locked in stable superstructures up to 40 °C because the CD signal never recovers fully during the thermal denaturation. Reannealing the sample from 55 °C, however, restores triple helicity to its original levels, in perfect agreement with rheology data. These results suggest that a reorganization of OKD self‐assemblies, rather than changes to their triple‐helical structure, is responsible for the elevated thermostability observed after deformation.

## 3 Discussion

Symmetrically designed 33‐mers based on the 11/3sb template reliably form nanofibers, making them the shortest CMPs to support sticky‐ended self‐assembly. Additionally, results from 11/3sb permutants exhibit many parallels with those from earlier 42‐residue designs.^[^ ^16^ ^]^ Despite differences in size, residue composition, and charge distribution, all 11/3sb and 14/4sb peptides form soluble, fibrillar self‐assemblies that show high triple‐helix content and cooperative denaturation curves via CD spectroscopy. Both systems are equally sensitive to disruptions to the salt‐bridge network that underlies their sticky‐ended association. Most interestingly, the thermostability of permutants follows the same order in both systems, suggesting that similar factors govern their self‐assembly. This observed consistency presents SESSA as a robust strategy for synthetic collagen production.

SESSA promises triple‐helical nanofibers with fully paired Lys and Asp residues. This design also reduces the availability of potential inter‐triple‐helical contacts necessary for a higher‐order association. The high propensity of 11/3sb permutants, such as OKD, to form inter‐triple‐helical associations and hydrogels is, therefore, especially surprising. Incomplete salt‐bridge formation could produce such contacts but would significantly compromise thermostability (Figure 4).^[^ ^22^ ^]^ Additionally, the thermostability of the 11/3sb and 14/4sb permutants display parallel trends irrespective of their tendency for a higher‐order association, indicating that these attributes are mostly independent. Thus, other moieties, such as terminal charges, are more likely to serve as inter‐triple‐helical contacts that facilitate higher‐order association.

Despite many strong parallels between 11/3sb and 14/4sb self‐assembly, the nanoscale organization and the thermostability of the nanofibers that they form present opposite trends. 11/3sb assemblies form increasingly complex structures with higher thermostability: DOK (*T*_m_ = 17 °C) nanofibers are thin and short, KDO (27 °C) exhibits increased bundling, and OKD (32 °C) associates into needles that assemble further into thick and extremely long nanofibers. In contrast, the thermostability of 14/4sb permutants tracks with a *lack of* higher‐order association.^[^ ^16^ ^]^ In both cases, the most thermostable nanofibers display bundling behaviors that are consistent with the structural patterns predicted by SESSA: 11/3sb series has easier access to bundle formation than 14/4sb does (Figure 2). The idealized molecular structures SESSA predicts are most accurate when self‐assemblies are highly thermostable (e.g., OKD permutants). The adherence of less stable nanofibers (e.g., KDO and OKD) to the idealized models, however, would diminish with decreasing thermostability. This dichotomy is especially apparent in the nanostructure of 11/3sb and 14/4sb DOK nanofibers, which clearly oppose the trends set by their OKD counterparts (Figure 5).^[^ ^16^ ^]^ Notably, such analyses are possible only because SESSA is able to offer a molecular understanding of the assembled state for synthetic collagens, something that is most often unavailable.

As with F0, 11/3sb‐OKD self‐assembly is highly hierarchical, presenting triple helices, needles, fibrils, and—ultimately—a hydrogel. There are, however, few additional similarities between the two systems. The relationship between the triple helicity and material properties of F0 self‐assemblies is complex. F0 hydrogels gradually lose triple‐helical structure above 20 °C, yet their *G*′ and *G*′′ rise in this range.^[^ ^13^ ^]^ High concentrations of F0 (>0.2% v/w) produce higher thermostability (*T*_m_ = 40 °C) than do low concentrations (<0.1% w/v; *T*_m_ = 25 °C), though without a marked increase in triple helicity.^[^ ^16^ ^]^ Finally, circular permutations of F0 do not self‐assemble, contrary to what is expected of CMPs that adhere to sticky‐ended association (Figure 1B).^[^ ^17^ ^]^ In contrast, the properties of 11/3sb‐OKD hydrogels can directly be linked to their triple‐helical structure. Their thermostability is not sensitive to peptide concentration, and there is a remarkable correspondence between denaturation curves obtained with OKD solutions (0.06% w/v) and hydrogels (0.5%) via both CD spectroscopy (Figures 3B and 4B) and rheometry (0.5% and 1% w/v) (Figure 6B). Even when hydrogel morphology is affected by high stress, a loss of triple‐helical structure and material stiffness accompany each other (Figure 7). Further, the thermostability of triple‐helical assemblies in high ionic strength (*I*  =  200 mm) phosphate buffer mirrors exactly the thermostability of OKD hydrogels in a cell‐culture medium (Figure S6, Supporting Information), pointing to ionic strength as the defining factor influencing hydrogel stability in biological media and marking a need for longer, symmetrically designed CMPs (≥42 residues).^[^ ^16^ ^]^ Finally, the rapid recovery of OKD gels after stress and their lower storage modulus as compared to F0 gels are both consistent with OKD nanostructure, which portrays a network formed by the association of needles into fibrils. Overall, the overwhelming consistency between solution‐phase and hydrogel data presents a clear link between triple‐helical structure and 11/3sb‐OKD self‐assembly and hydrogelation, which has not been demonstrated previously.

## 4 Conclusion

The design of CMPs that self‐assemble through sticky ends is complicated by constraints imposed by the triple‐helical architecture of collagen. The 11/3sb permutants that arise from SESSA are the shortest CMPs to associate with sticky ends to form stable triple‐helical nanofibers, as well as hydrogels, and exhibit remarkable agreement between their molecular models, physicochemical properties, and materials characteristics. This ability to relate atomic models to higher‐order structure is especially critical for the rational design of synthetic collagen hydrogels for specific applications.^[^ ^23^ ^]^ As a robust design methodology, SESSA holds the potential to bring tailored synthetic collagen bio‐ and nanomaterials within reach.

## 5 Experimental Section

### Peptide Synthesis

CMPs were synthesized on glycine‐preloaded Wang or polyethylene glycol‐based resins using Fmoc‐protected monomers from Chem‐Impex, as described previously.^[^ ^19^ ^]^ Condensation of Fmoc‐ProHypGly‐OH,^[^ ^11b^ ^]^ Fmoc‐GlyProHyp‐OH (Bachem), or Fmoc‐GlyPro‐OH (Chem‐Impex) segments was employed wherever applicable. Isolated crude peptides were purified, as described previously,^[^ ^16^ ^]^ to >95% purity according to analytical HPLC and MALDI–TOF mass spectrometry (MS). MS analyses were carried out on an Applied Biosystems Voyager DE‐Pro mass spectrometer at the University of Wisconsin–Madison Biophysics Instrumentation Facility (UW–BIF; www.biochem.wisc.edu/bif). *m*/*z* calcd for 11/3sb variants [M + H]^+^, 3057.2; found, 3057.1 for OKD; found, 3057.4 for KDO; found, 3057.0 for DOK; found, 3057.3 for DOKctrl. Analytical HPLC results for purified CMPs are shown in Figure S1 (Supporting Information). *m*/*z* calcd for 8/2sb‐OKD [M + H]^+^, 2222.3; found, 2222.4. Analytical HPLC chromatograms of purified peptides are presented in Figures S1 and S2A (Supporting Information).

### Sample Preparation

All peptide solutions were prepared in 10 mm sodium phosphate buffer, pH 7.0 (*I* = 20 mm), unless noted otherwise. The addition of NaCl to 180 mm yielded high ionic‐strength samples (*I* = 200 mm) for CD analysis. To ensure the formation of the thermodynamic product, all peptide solutions were annealed from 55–4 °C at a rate of −12 °C/h and left at 4 °C for ≥48 h before data acquisition. Hydrogels were annealed at 20 °C and retained there for 10 h prior to rheological analysis.

### Circular Dichroism Spectroscopy

CD data on 0.6 mg/mL (≈0.2 mm) peptide samples was collected at the UW–BIF, as described previously.^[^ ^16^ ^]^ Data on 11/3sb‐OKD hydrogels were collected at the Massachusetts Institute of Technology Biophysical Instrumentation Facility (MIT–BIF; csbi.mit.edu/instrumentation) on a JASCO Model J‐1500 spectrophotometer in 0.1‐cm path‐length quartz cuvettes. Spectra were recorded at 4 or 20 °C with a 1 ‐nm band‐pass filter and an averaging time of 8 s. For thermal denaturation experiments, the CD signal at 226 nm was monitored while heating the sample in 1‐ °C steps at 12 °C/h. The denaturation transition is indicated by a minimum on the temperature‐derivative of the denaturation curve (∂[*θ*]/∂T). *T*_m_ values were determined assuming a constant curvature in the immediate vicinity of this minimum.

### Analytical Ultracentrifugation

Sedimentation equilibrium experiments were performed at the UW–BIF with a Beckman Coulter XL‐A analytical ultracentrifuge in accordance with prior work.^[^ ^16^ ^]^ Experiments were run at 4 °C on 0.3‐mg/mL peptide solutions for 8 days at speeds of 8000, 12 000, 18 000, 28 000, 38 000, and 48 000 rpm. Equilibrium gradients were modeled as single and multiple noninteracting species using a buffer density of 1.0011 g/mL and a partial specific volume of 0.69458 mL/g, as calculated based on amino acid content.^[^ ^24^ ^]^ Nonsedimenting baselines at 0.03–0.05 attenuance were applied for all samples during analysis, which was performed with programs written by D. R. McCaslin (UW–BIF) for IGOR PRO (WaveMetrics). The best fits of gradient data to monomer + multimers models at 18 000 rpm are shown in Figure S4 (Supporting Information).

### Transmission Electron Microscopy

All TEM imaging was performed at the UW–Madison Nanoscale Imaging and Analysis Center (wcnt.wisc.edu/center‐for‐nanoscale‐imaging‐and‐analysis) with a FEI Tecnai T12 TEM instrument (120 kV). Quantifoil R1.2/1.3 holey–carbon mesh on copper TEM grids was pretreated by glow discharge for 0.5 min at 25 mA with a Pelco easiGlow unit from Ted Pella. Lacey carbon/Formvar TEM grids were used as purchased. Filtered samples were allowed to adhere to the grids at 4 °C for 1 min. Excess liquid was blotted with filter paper, and the grid was allowed to dry for 5 min. A negative stain was applied for 10 min by inverting the grid on a drop of a freshly prepared solution of phosphotungstic acid (2.0% w/w) that was adjusted to pH 6.0 with aqueous NaOH and filtered. Excess stain was removed with filter paper, and the grid was allowed to dry overnight prior to imaging.

### Rheology

All rheological studies were performed with an Anton–Paar 702 rheometer in single‐drive mode using a cone‐plate geometry (CP25‐1/S; 25‐mm diameter; 1° rise). Peptide solutions were heated above 45 °C to eliminate any gelation present therein and applied to the stage immediately. Samples were then allowed to anneal from 55 to 20 °C on the stage. Edges of the sample‐plate interface were sealed using mineral oil to minimize evaporation. Data for strain (at 3 rad/s) and frequency sweeps (at 1% strain) were collected at 20 °C after 10 h at that temperature; temperature response (at 3 rad/s and 1% strain) was determined between 20 and 55 °C. Endothelial Cell Growth Basal Medium 2 (EBM‐2; Lonza) was employed when evaluating the 11/3sb‐OKD hydrogels in cell culture medium (Figure S6, Supporting Information).

### Computational Modeling and Analysis

Structural models of CMP self‐assembly were built using Gencollagen (UCSF; www.rbvi.ucsf.edu/cgi‐bin/gencollagen.py) and analyzed with PyMOL v1.8 (Schrödinger). Association states for sticky‐ended assemblies (Figure 3A) were enumerated and strand‐association landscapes were constructed using purpose‐written Python (v.2.7) scripts.

## Conflict of Interest

The authors declare no conflict of interest.

## Supporting information

Supporting Information

## Data Availability

The data that support the findings of this study are available in the supplementary material of this article.
