# Supplementary material for: Synthetic Collagen Hydrogels through Symmetric Self‐Assembly of Small Peptides
Source: Adv Sci (Weinh). 2023 Nov 23;11(3):2303228. doi: 10.1002/advs.202303228 (PMC10797479; doi:10.1002/advs.202303228)
Supplement: Supplementary file 1 — Supporting Information [file ADVS-11-2303228-s001.pdf]

ADVANCED SCIENCE
Open Access

Supporting Information

for *Adv. Sci.*, DOI 10.1002/advs.202303228

Synthetic Collagen Hydrogels through Symmetric Self-Assembly of Small Peptides

*I. Caglar Tanrikulu, Lianna Dang, Lekha Nelavelli, Aubrey J. Ellison, Bradley D. Olsen, Song Jin and Ronald T. Raines**

Synthetic collagen hydrogels through symmetric self-assembly of small peptides

*I. Caglar Tanrikulu, Lianna Dang, Lekha Nelavelli, Aubrey J. Ellison, Bradley D. Olsen, Song Jin and Ronald T. Raines**

Content		Page
Table of Contents		S1
**Figure S1.**	Analytical HPLC chromatograms of purified 11/3sb-derived CMPs	S2
**Figure S2.**	Purification and self-assembly of 8/2sb-OKD	S3
**Figure S3.**	Thermal denaturation curves for 11/3sb-OKD and -KDO at high and low ionic strength	S4
**Figure S4.**	Sedimentation equilibrium analysis of 11/3sb-DOK and -DOKctrl assembly	S5
**Figure S5.**	Rheology of 0.5% w/v 11/3sb-OKD hydrogels	S6
**Figure S6.**	Rheology of 1.0% w/v 11/3sb-OKD hydrogels in cell-culture medium (EBM-2)	S7
**Figure S7.**	Heating 11/3sb-OKD hydrogels to 45 °C and subsequent cooling does not remove the second thermal transition in rheology	S8

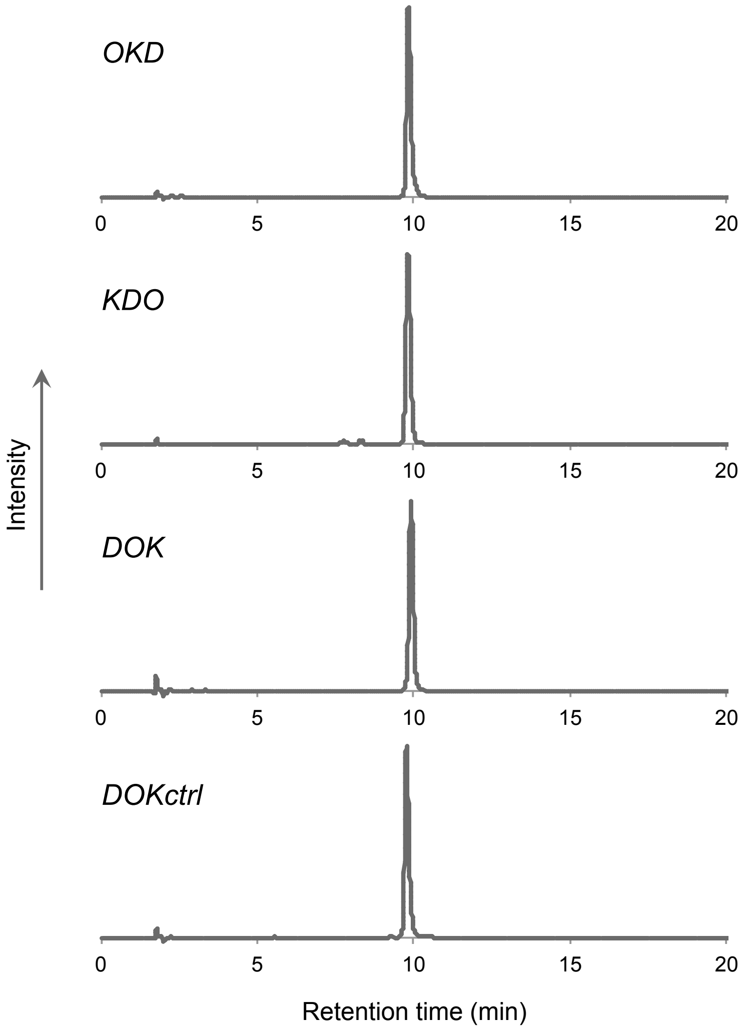

Figure S1. Analytical HPLC chromatograms of purified 11/3sb-derived CMPs. Chromatograms were acquired on a VarioPrep 250/4.6 C18 analytical column from Macherey–Nagel with a 6–28.5% v/v acetonitrile gradient in water over 15 min at a flow rate of 1.5 mL/min.

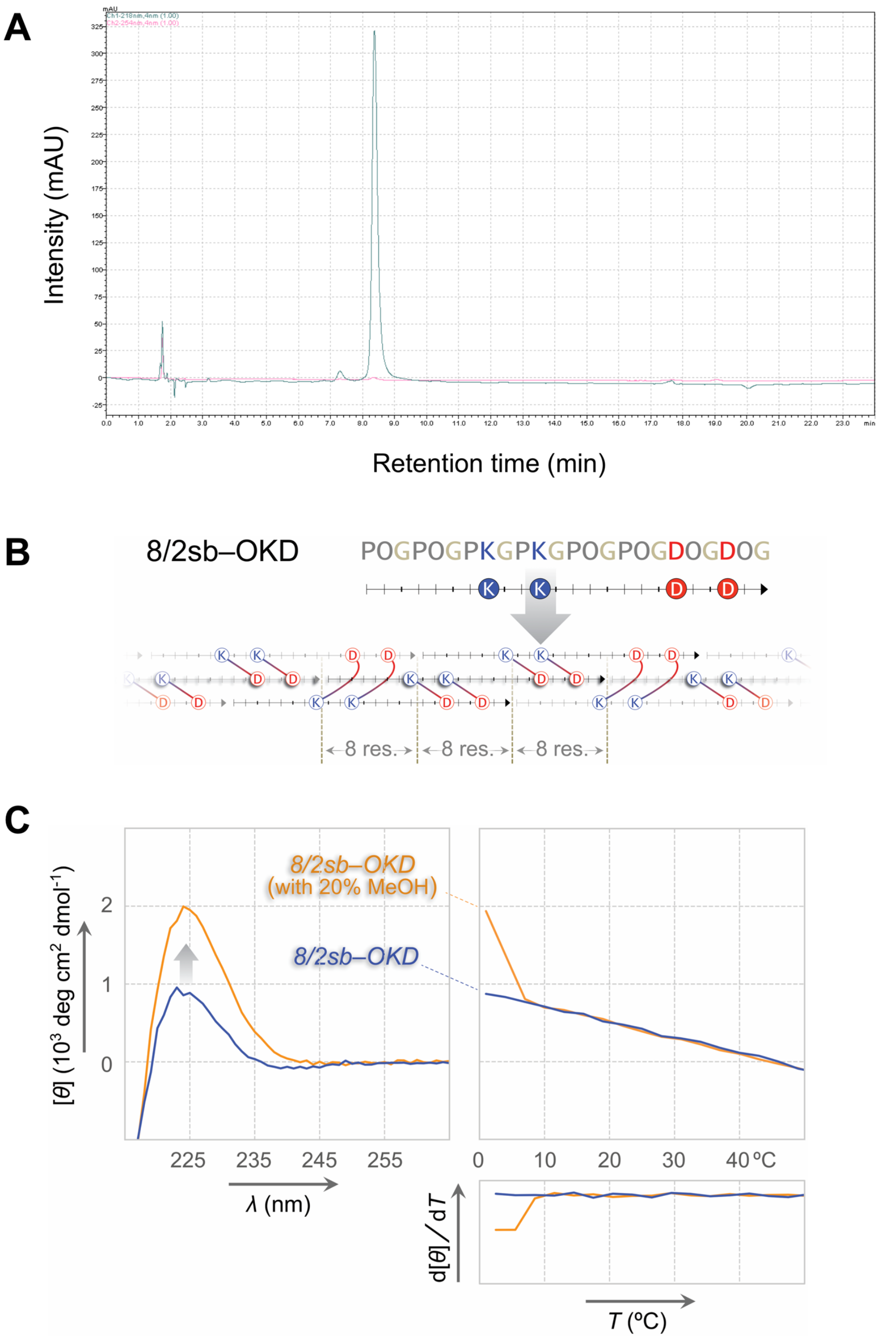

Figure S2. Purification and self-assembly of 8/2sb-OKD. (A) Analytical HPLC chromatogram of purified 8/2sb-OKD acquired on a Macherey–Nagel VarioPrep 250/4.6 C18 column on a 9–21% v/v acetonitrile gradient in water over 14 min at a flow rate of 1.5 mL/min. (B) Sticky-ended symmetric self-assembly of 8/2sb-OKD. (C) Circular dichroism spectra (at 1 °C) and thermal denaturation profiles for 8/2sb-OKD at 1.5 mg/mL concentration. Addition of 20% v/v methanol leads to triple-helix formation at ~0 °C.

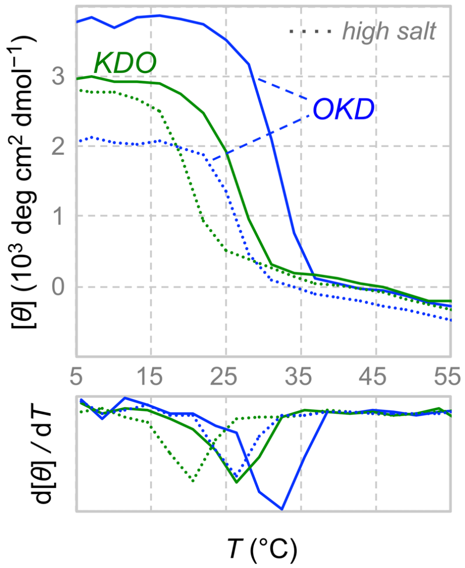

Figure S3. Thermal denaturation curves for 11/3sb-OKD and -KDO at high and low ionic strength. Data is shown at I = 20 mM (lines) and 200 mM (dotted lines). Thermostability and structure content for both CMPs diminish at high salt concentrations.

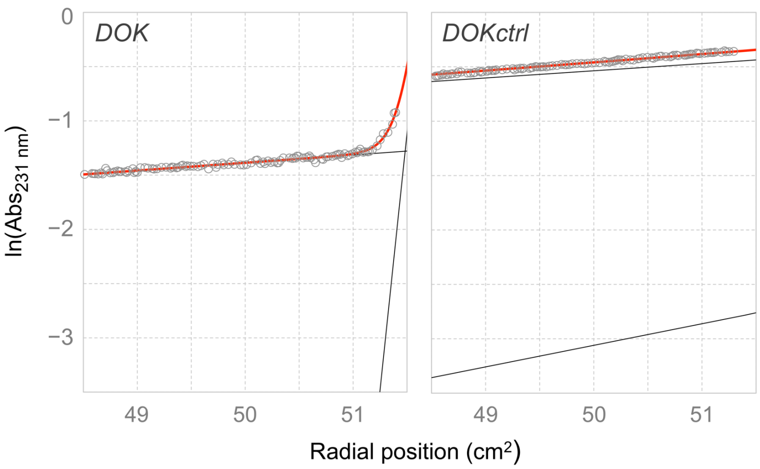

Figure S4. Sedimentation equilibrium analysis of 11/3sb-DOK and -DOKctrl assembly. Equilibrium gradients at 18000 rpm (circles) are shown with models (red lines) that provide optimal fits to gradient data. Models feature a monomeric and a multimeric component (black lines). The DOK data (left) is best explained by a monomer + 132-mer mixture. DOK forms large assemblies, and most of the peptide settles to the bottom of the cell as evidenced by a sharp spike in absorbance there. The gradient shifts significantly with increased speed, and therefore, a model based on data acquired at only a single speed (18000 rpm) is shown. In contrast, the DOKctrl gradient (right) is best represented as a monomer + trimer mixture, with the monomeric component dominating the gradient. The model featured here was built on the complete set of data, recorded at six speeds ranging from 8000 to 48000 rpm.

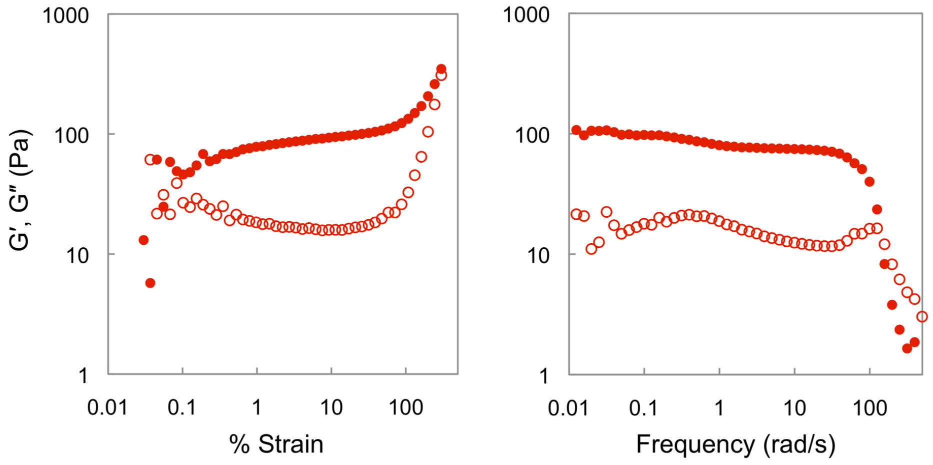

Figure S5. Rheology of 0.5% w/v 11/3sb-OKD hydrogels. Gels were cast on stage in 10 mM sodium phosphate buffer, pH 7.0, and strain (at 3 rad/s) and frequency sweeps (at 1% strain) were performed. Closed (●) and open circles (○) report storage and loss moduli.

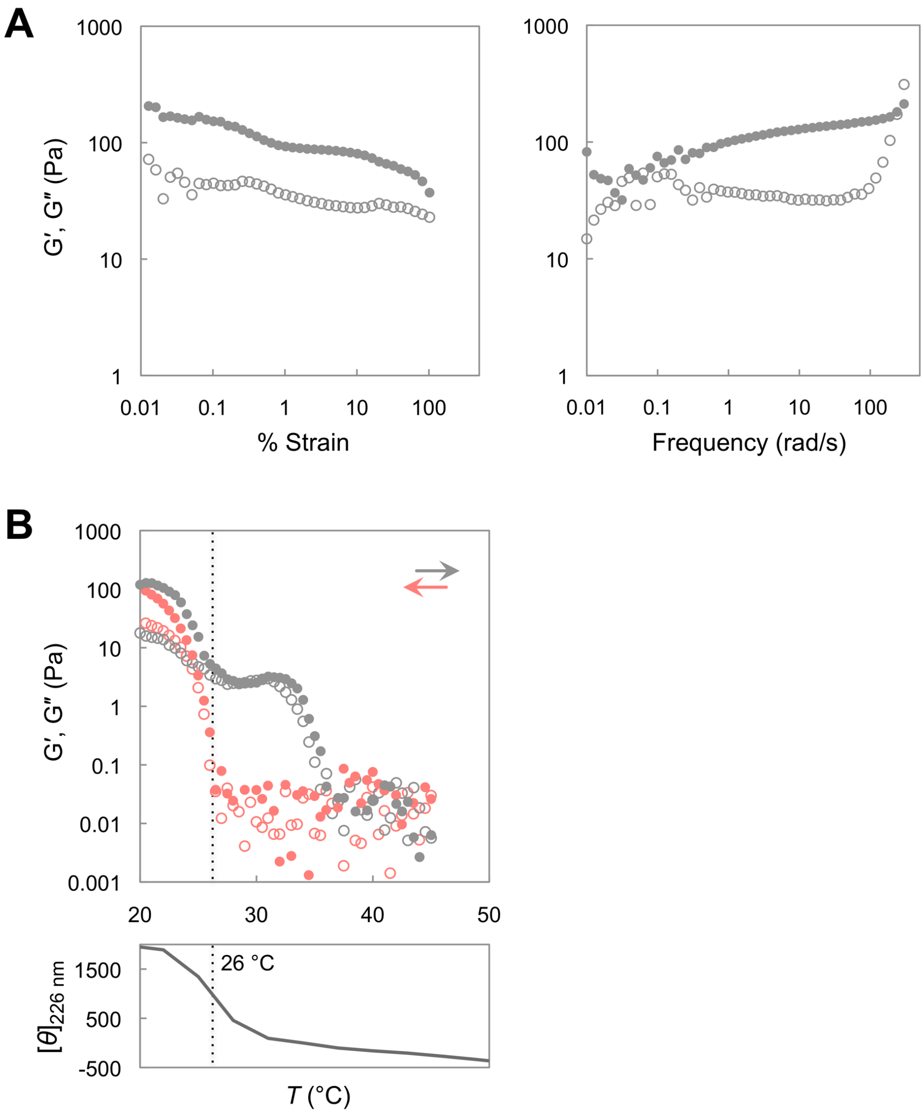

Figure S6. Rheology of 1.0% w/v 11/3sb-OKD hydrogels in cell culture medium (EBM-2). (A) Strain (at 1 rad/s) and frequency sweeps (at 1% strain). (B) Temperature denaturation (gray) and subsequent annealing (red) of a 1.0% w/v OKD hydrogel in EBM-2. Despite a complex denaturation, cooling the peptide solution through 26 °C, the T_m value for OKD in sodium phosphate buffer, pH 7.0, at I = 200 mM (bottom panel and dashed line), restores the hydrogel. Closed (●) and open circles (○) report storage and loss moduli.

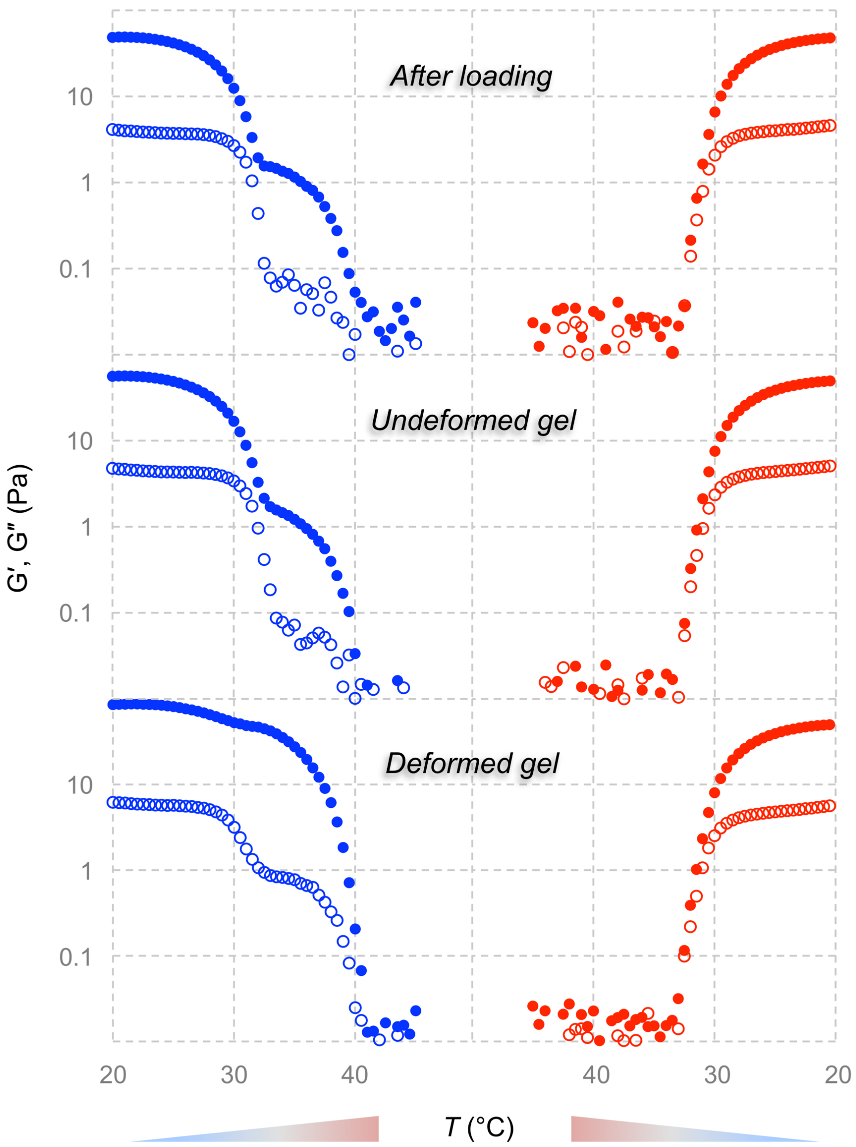

Figure S7. Heating 11/3sb-OKD hydrogels to 45 °C and subsequent cooling does not remove the second thermal transition in rheology. Temperature denaturation to 45 °C (blue) and subsequent annealing (red) of a 0.5% w/v 11/3sb-OKD hydrogel is shown following sample loading (top), immediately after initial annealing (middle), and after experiencing deformation immediately after the second annealing step. Two-stage denaturation of the hydrogel is apparent in the middle panel, despite no history of deformation following annealing from 45 °C. After being stressed to 500%, both the denaturation transition at 40 °C and the elevated moduli become more apparent (bottom panel). Closed (●) and open circles (○) report storage and loss moduli.
